# Double valve replacement in a patient with Maroteaux – Lamy syndrome as an ultimate team challenge

**DOI:** 10.1186/s13019-021-01530-x

**Published:** 2021-05-24

**Authors:** Alexandros Agron Demis, Stella Oikonomidou, Fotios Daglis, Spyridon Polymenakos, Matthew Panagiotou

**Affiliations:** 1 Cardiovascular Surgery Department, Athens Medical Center, Athens, Greece; 2 Anesthesiology Department, Athens Medical Center, Athens, Greece; 3 Pathology Department, Athens Medical Center, Athens, Greece

**Keywords:** Maroteaux-Lamy syndrome, Mucopolysaccharidosis, Heart valve surgery, Case report

## Abstract

**Background:**

The Maroteaux-Lamy syndrome (Mucopolysaccharidosis type VI) is a rare, inherited metabolic disease that results in progressive tissue accumulation of dermatan-sulfated glycosaminoglycans and inflammatory consequences that almost always affects the heart valves. From the anesthesia point of view, managing the airway and ventilation might be a serious challenge due to specific features of the syndrome. Additionally, it is more than probable that the surgical team will perform a non-straightforward procedure.

**Case presentation:**

A 42-year-old male with Maroteaux-Lamy syndrome was referred to our department with shortness of breath, due to severe aortic stenosis, and at least moderate mitral valve regurgitation.

The patient was initially scheduled for aortic valve replacement. After multiple attempts with video assisted laryngoscopy, the endotracheal intubation was achieved with the aid of fiberoptic bronchoscopy, while the ventilation succeeded only with laryngeal mask. The somatic features of the syndrome that made the anesthesia induction extremely difficult, also affected the surgical procedure. Suboptimal exposure of the mitral valve, patch enlargement of the aortic root to host the bigger possible prosthesis, and the hard decision to replace the mitral valve even with a marginal indication were the intraoperative challenges for the surgical team.

Finally, the patient underwent a successful double valve replacement with aortic root enlargement and 18 months postoperatively remains improved.

**Conclusion:**

Patients with Maroteaux-Lamy syndrome represent a challenge for both anesthesiologists and cardiac surgeons. The whole team should be well prepared to deal with difficulties in airway management, ventilation and surgical valve exposure. The cardiac surgeon should be ready to offer additional procedures and even replace “prematurely” a moderately diseased valve in order to avoid a dangerous reoperation. The limited knowledge on the natural history of the Maroteaux-Lamy syndrome valvulopathy and the difficulties in anesthesia induction support this approach.

## Background

Maroteaux-Lamy syndrome, first described by Maroteaux in 1963, is a rare, inherited metabolic disease, caused by mutations in the gene coding for N-acetylgalactosamin-4-sulfatase, a lysosomal enzyme involved in the degradation of dermatan sulfate [1]. Dermatan-sulfated glycosaminoglycans are a prominent component of normal cardiac valve tissue, so their progressive accumulation and inflammatory consequences result in left heart valve degeneration. Nearly all the patients with Maroteaux-Lamy syndrome will develop cardiac valve thickening, and dysfunction requiring valve replacement during their life course [2]. It is more than probable, that the procedure will not be straightforward due to the physical characteristics of these patients. In the literature, there are only few reports of double valve replacement in patients with this syndrome. We present our experience highlighting the intraoperative management, and decision making.

## Case presentation

**Fig. 1:**
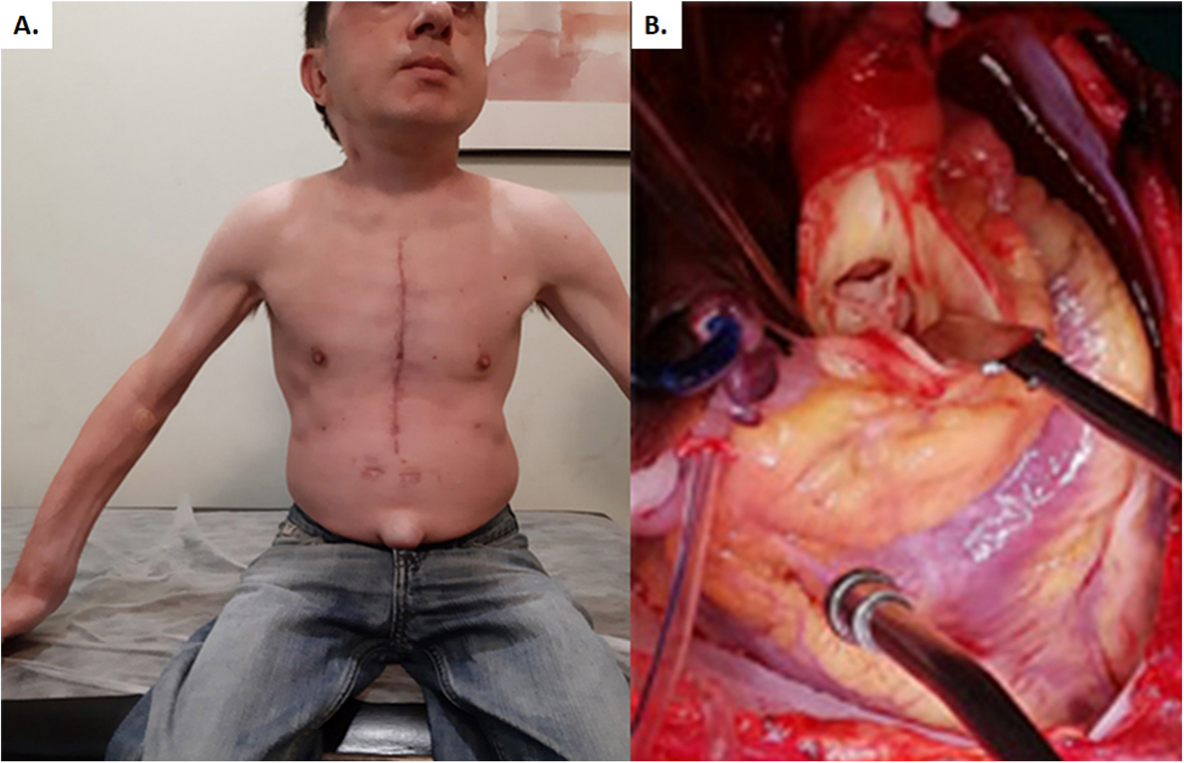
**a**. Fibrotic and stenotic aortic valve. **b**. The postoperative photo of the patient shows his phenotype

A 42-year-old male with diagnosed Maroteaux-Lamy syndrome since childhood was referred to us due to symptomatic severe aortic stenosis, along with at least moderate mitral valve regurgitation. The patient was presented, in sinus rhythm with normal cognitive function, short trunk (H: 135 cm, W: 40 kg), stiff neck, limited joints mobility, hypertrophic tongue, forehead bossing, limited mouth opening, reduced visual acquity, and umbilical hernia (Fig. 1a). The echocardiogram showed severe aortic valve stenosis (mean gradient 69 mmHg, peak velocity 4,16 m/s), at least moderate mitral regurgitation (regurgitant orifice area 0.37 cm^2^, regurgitant fraction 50%), normal tricuspid and pulmonary valve function, left ventricular hypertrophy (mass index 120 g/m^2^), left ventricle ejection fraction 55%, indexed left atrial volume 35 ml/m^2^, and mean pulmonary artery systolic pressure at rest 28 mmHg. The angiogram revealed normal coronary arteries.

The oropharyngeal features such as neck stiffness and small mouth opening with hypertrophic tongue, made the airway management a very difficult and life-threatening procedure. Specifically, the routine endotracheal intubation was impossible, despite the multiple attempts even with video assisted laryngoscopy. Meanwhile, the ventilation was limited by the restricted compliance of the thoracic cage due to the thoracic joints degeneration and stiffness, and was possible only with the aid of laryngeal mask. Emergency cricothyroidotomy was held as a bailout option due to the difficulty of the neck extension and the possible interference with the median sternotomy. Moreover, it is important to avoid tracheal and cartilaginous tissues manipulation in these patients since they might result in tracheomalacia. Finally, the intubation was achieved with fiberoptic bronchoscope. The insertion of a transesophageal ultrasound probe was considered dangerous and therefore was avoided.

The operation was performed with midline sternotomy, aorto-bicaval cannulation, moderate hypothermia and Bretschneider cardioplegia. The interatrial approach with careful dissection of the interatrial groove, mobilization of both vena cava from the pericardial tissue and wide opening of the left mediastinal pleura to facilitate heart rotation, were proved to be adequate additional maneuvers for the exposure of the mitral valve. No extension of the atriotomy through the right atrium was necessary.

The intraoperative findings included fibrotic and stenotic aortic valve (Fig. 1b) with a small aortic root and macroscopic degeneration of mitral leaflets. An aortic and mitral valve replacement with Sorin Bicarbon Slimline 17 and Sorin Bicarbon Fitline 23 (Sorin Group Italia S.r.l., Saluggia VC Italy) respectively was performed. The decision to replace the mitral valve was imposed by the macroscopical appearance of the leaflets, the degree of moderate regurgitation, the uncertain natural history of this specific valve disease with the possibility of another dangerous anesthesia induction in the future. The small aortic ring and aortic root were addressed with a modified Nick’s procedure using a polyester vascular patch (LeMaitre Vascular, Burlingtone, MA USA).

**Fig. 2:**
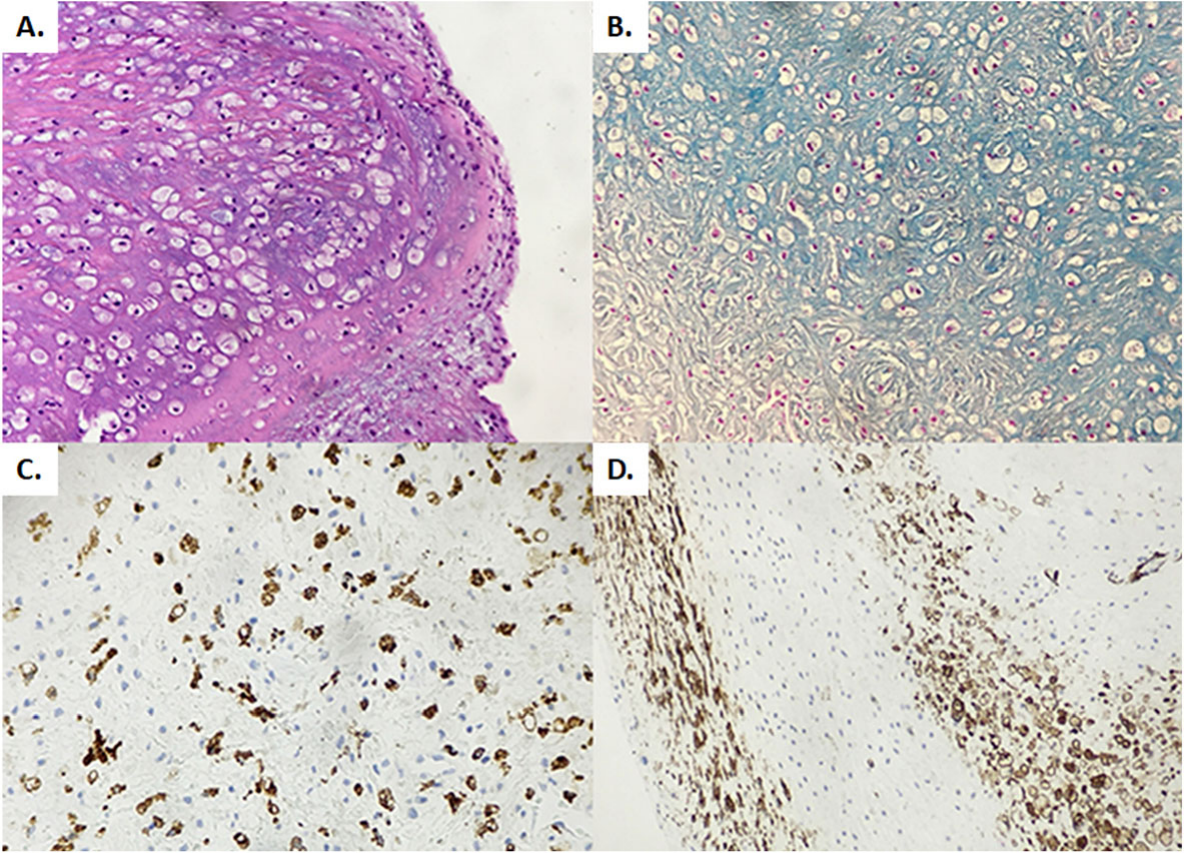
Mitral valve histological examination. **a**. Valve stromal infiltration from clear cells, polysaccharides and mucopolysaccharides, **b**. Alcian Blue 2,5 positive in stromal glycosaminoglycans, **c.** CD68 (+) in clear cells, **d**. Actin (+) in myofibroblasts and clear cells

The patient experienced an uneventful in hospital course, and he was discharged the 8th postoperative day. No patient-prosthesis mismatch signs were presented. The histological examination of the mitral valve revealed leaflets myxoid degeneration and clear cells accumulation (Fig. 2). Eighteen months postoperatively, the patient remains asymptomatic in sinus rhythm (Fig. 1a). The recent echocardiogram showed normal left ventricular function, normal function of the prosthetic valves without leak. The aortic prosthesis mean and peak gradients were 11, 8 and 22, 8 mmHg respectively, and the aortic peak velocity 2, 39 m/sec. The mitral prosthesis peak gradient was 6, 63 m/sec.

## Discussion and conclusions

The Maroteaux-Lamy syndrome prevalence varies among populations from 0.0132 to 20.0/100000 newborns [1]. Despite the heterogeneity of the syndrome phenotype, mental development is usually normal, and cardiac valve pathology is present in almost all individuals. Somatic features of the syndrome are growth retardation, dwarfism, coarse facial features, joint stiffness, dysostosis multiplex, hepatosplenomegaly, corneal clouding, macrocephaly, hearing loss, and umbilical, or inguinal hernias [2]. Specific features of the syndrome constitute a great challenge for both anesthesiologists and cardiac surgeons. Neck stiffness, large tongue, small mouth opening, tracheobronchial narrowing, chest deformities and poor ribs mobility, all represent challenges for airway management and ventilation. Fatal events during anesthesia induction have been described in the literature [3].

The difficulty of sternal retraction due to skeletal abnormalities and consequently problematic mitral valve exposure, along with the inconveniently small aortic and mitral annulus with increased patient-prosthesis mismatch possibility, constitute the intraoperative challenges for the cardiac surgeon.

In the literature, bail out maneuvers like implanting a reversed aortic prosthesis in the mitral position or aortic root enlargement, such as the modified Nick’s we offered to our patient are extremely rare [4].

In addition to the technical difficulties, hard intraoperative decisions will be taken, especially in cases of a second moderately diseased valve, such as the mitral valve of our patient. Performing a double valve replacement increases the operative risk. Leaving unaddressed the second diseased valve exposes the patient to an increased risk of a difficult and dangerous reoperation. Previous papers proposed a non-aggressive approach with the second diseased valve, expecting stabilization or improvement of the valve disease with the enzyme replacement therapy [5]. Unfortunately, the enzyme replacement therapy despite the positive effect on myocardial hypertrophy and ejection fraction, it has no clear effect on the valve disease which should be considered as irreversible and progressively worsening. A possible explanation might be the suboptimal bio-distribution of the enzyme therapy in the valve tissue. For the same reason bone and articular cartilage disease in these patients are not reversed or even stabilized with enzyme replacement therapy [6].

The need to avoid a dangerous reoperation should influence the decision regarding the second diseased valve.

In conclusion, patients with Maroteaux-Lamy syndrome represent a heart team challenge. Proper cardiac anesthesia and cardiac surgery preparation are mandatory for the management of such a rare and vulnerable patient. An additional “premature” replacement of a second moderately diseased valve might be a useful option.

## Data Availability

The used data sets of the current study are available from the corresponding author on reasonable request.

## References

[1] Tomanin R, Karageorgos L, Zanetti A, al-Sayed M, Bailey M, Miller N, Sakuraba H, Hopwood JJ (2018). Mucopolysaccharidosis type VI and molecular analysis: review and classification of published variants in the ARSB gene. Hum Mutat.

[2] Braulin E, Harmatz P, Scarpa M (2011). Cardiac disease in patients with mucopolysaccharidosis: presentation, diagnosis and management. J Inherit Met Dis.

[3] Tan CC, Schaff HV, Miller FA (1992). Valvular heart disease in four patients with Maroteaux – Lamy syndrome. Circulation.

[4] Bell DJW, He C, Pauli JL, Naidoo R. Maroteaux-Lamy syndrome: a rare and challenging case of mitral valve replacement Asian. Cardiovasc Thorac Ann, 2016. 2018;26(7, Oct 18):560–2. 10.1177/0218492316675533.Epub. 10.1177/0218492316675533 30253663

[5] Torre S, Scarpelli M, Salviati A, Buffone E, Faggian G, Luciani GB (2016). Aortic and mitral valve involvement in Marroteaux-Lamy syndrome VI: surgical implications in the enzyme replacement therapy era. Ann Thorac Surg.

[6] Concolino D, Deodato F, Parini R (2018). Enzyme replacement therapy: efficacy and limitations. Ital J Pediatr.

